# β-Caryophyllene Inhibits Cell Proliferation through a Direct Modulation of CB2 Receptors in Glioblastoma Cells

**DOI:** 10.3390/cancers12041038

**Published:** 2020-04-23

**Authors:** Natasha Irrera, Angela D’Ascola, Giovanni Pallio, Alessandra Bitto, Federica Mannino, Vincenzo Arcoraci, Michelangelo Rottura, Antonio Ieni, Letteria Minutoli, Daniela Metro, Mario Vaccaro, Domenica Altavilla, Francesco Squadrito

**Affiliations:** 1 Department of Clinical and Experimental Medicine, University of Messina, c/o AOU Policlinico G. Martino, Via C. Valeria Gazzi, 98125 Messina, Italy; nirrera@unime.it (N.I.); adascola@unime.it (A.D.); gpallio@unime.it (G.P.); abitto@unime.it (A.B.); fmannino@unime.it (F.M.); varcoraci@unime.it (V.A.); rotturamichelangelo@gmail.com (M.R.); lminutoli@unime.it (L.M.); vaccaro@unime.it (M.V.); 2 Department of Human Pathology in Adult and Developmental Age “Gaetano Barresi”, University of Messina, c/o AOU Policlinico G. Martino, Via C. Valeria Gazzi, 98125 Messina, Italy; aieni@unime.it; 3 Department of Biomedical and Dental Sciences and Morphofunctional Imaging, University of Messina, c/o AOU Policlinico G. Martino, Via C. Valeria Gazzi, 98125 Messina, Italy; dmetro@unime.it (D.M.); daltavilla@unime.it (D.A.)

**Keywords:** glioblastoma, β-caryophyllene, CB2 receptor, apoptosis, inflammation

## Abstract

Glioblastomas are aggressive cancers characterized by uncontrolled proliferation and inflammation. b-caryophyllene (BCP) is a cannabinoid receptor 2 (CB2) agonist that showed an important anti-inflammatory effect through the interaction of CB2 and peroxisome proliferator-activated receptor gamma (PPARg) receptors. BCP effects were investigated in an in vitro model of glioblastoma. U-373 and U87, derived from a human glioblastoma, and human glioma stem-like cells (GSCs) were treated with BCP at different doses and time-points. AM360, a specific CB2 antagonist, was added 2 h before BCP treatment. BCP showed a significant anti-proliferative effect, reducing cell viability, inhibiting cell cycle, and increasing apoptosis, as demonstrated by Tunel assay, caspase-3 and caspase -9 activation. In addition, the pro-apoptotic BAX expression was increased, whereas the anti-apoptotic Bcl-2 expression was reduced. Treatment with BCP decreased Beclin-1, LC3 and p62/SQSTM1 expression, indicating a possible switch of autophagy to apoptosis. BCP’s anti-inflammatory effect was demonstrated by NF-κB reduction, PPARg activation and TNF-a decrease; BCP significantly reduced Jun N-Terminal Kinase (JNK) expression as a consequence of TNF-α inhibition. AM360 abrogated BCP effects, thus demonstrating the BCP mechanism of action through the CB2 receptor. These findings let us hypothesize that BCP may act as a tumor suppressor in glioblastoma, acting on CB2 receptor and modulating JNK.

## 1. Introduction

Glioblastomas (GBMs) are malignant tumors of the central nervous system that may appear as de novo cancers, primary GBMs, or may develop from glioma [1]. GBMs are particularly aggressive cancers characterized by an exaggerated proliferation and uncontrolled angiogenesis, thus promoting tumor growth. Different types of cancers, including glioblastomas, showed the activation of Nuclear Factor kappa B (NF-κB) and mitogen-activated protein kinase (MAPK) pathways, which often stimulates cell growth and proliferation [2]. NF-κB is highly expressed in gliomas and may worsen the prognosis of the disease [3]. The transcription factor NF-κB regulates the expression of genes involved in inflammatory response, thus releasing pro-inflammatory cytokines [4]; increased levels of pro-inflammatory cytokines have also been shown in gliomas, which may be considered as a pro-inflammatory neoplasia [5]. 

Treatment of GBM is currently based on surgical removal followed by radiotherapy and the administration of temozolomide, as adjuvant therapy [6]. However, the median survival of patients is of 5 years following initial diagnosis. For this reason, new therapeutic strategies are necessary to improve the prognosis of the disease as well as the quality of life of patients. Cannabinoid specific receptors CB1 and cannabinoid receptor 2 (CB2), involved in the control of cell proliferation, differentiation and survival, are distributed in brain and are expressed in different cell types, such as astrocytes, microglia and glioblastoma cells [7]; therefore, targeting CB receptors might represent an interesting strategy. Cannabinoids are constituents of the plant Cannabis sativa and cannabis constituents have been used in traditional medicine thanks to their curative properties. Among them, phytocannabinoids, cannabinol, cannabidiol (CBD), cannabigerol or β-caryophyllene (BCP) [8,9] have been extracted to avoid psychoactive activity related to cannabinoid ∆9-tetrahydrocannabinol (THC). In vivo studies described the cannabinoids mechanism of action on cancers, indicating that CB agonists act (i) inducing cell death processes such as apoptosis and/or autophagy and (ii) inhibiting cell proliferation [10]. BCP is a bicyclic sesquiterpene whose possible efficacy has been yet described on cancer cells [11]. In addition, an in vivo study demonstrated that BCP treatment may modulate inflammation through a crosstalk between CB2 receptor and peroxisome proliferator-activated receptor gamma (PPAR-γ) [12]. However, the effects of BCP on glioblastoma have not been yet deeply investigated and the exact mechanism of action of BCP has to be deeply explained. Therefore, the aim of this study was to investigate BCP effects in an in vitro model of glioblastoma. 

## 2. Results

### 2.1. BCP Reduces Cell Viability

**Figure 1:**
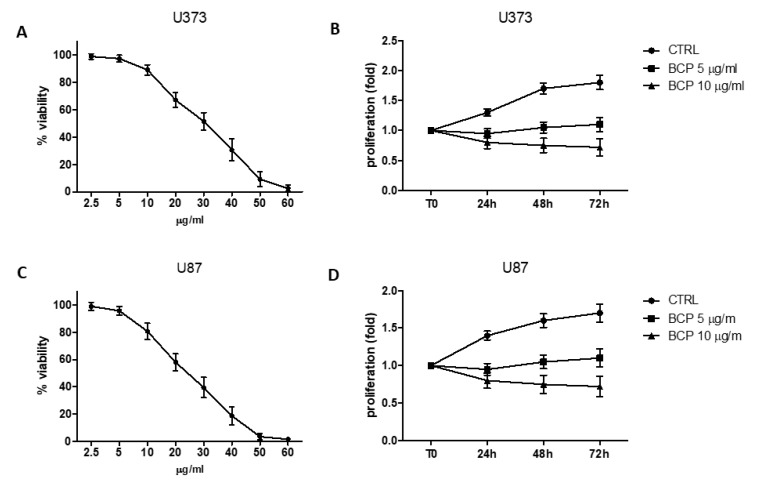
Dose response curve of cell viability assessed in U373 (panel **A**) and U87 (panel **C**) cells treated with b-caryophyllene (BCP). Values are expressed as percentage of reduction of viability with respect to the untreated control. Cell proliferation evaluation at T0, 24, 48 and 72 h in U373 (panel **B**) and U87 (panel **D**) cells treated with BCP. Values are expressed as fold change of viability with respect to the untreated control at T0.

BCP cytotoxicity was evaluated by incubating U373 with increasing concentrations, starting from 2.5 to 60 μg/mL. As shown in Figure 1 (panel A), a reduction in cell viability was observed when cells were treated with BCP concentrations up to 60 μg/mL. The most effective dose was 60 μg. Notably, cell viability was reduced to about 70% with 20 μg/mL and 50% with 30 μg/mL BCP at 24 h, respectively (Figure 1A). To examine the anti-proliferative effects, cells were treated with 10 or 20 μg/mL concentrations of BCP for indicated time intervals. The results of MTT (3-(4,5-dimethylthiazol-2-yl)-2,5-diphenyltetrazolium bromide) assay showed that BCP suppressed cell proliferation in these glioma cells in a dose- and time-dependent manner (Figure 1, panel B).

A p53 wild-type glioma cell line (U87) was also used to evaluate potential differences compared to U373 cells which are p53 mutant cell line. As shown in Figure 1 (panels C and D), BCP exhibited both cytotoxic and anti-proliferative effects in these glioma cells, resulting effective at slightly lower doses. Indeed, cell viability was reduced to about 60% with 20 μg/mL and 40% with 30 μg/mL BCP at 24 h and 5 μg/mL BCP was able to suppress cell proliferation until 72 h.

### 2.2. BCP Induces Apoptotic Cell Death 

**Figure 2:**
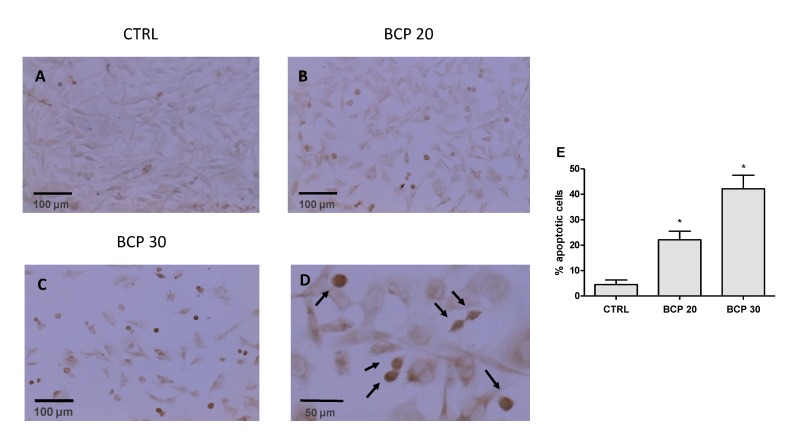
Effect of BCP on apoptosis induction in U373 cells. The detection of apoptosis was assessed by TUNEL (Terminal deoxynucleotidyl transferase dUTP nick end labeling) assay. In the images (panels **A**–**C**; scale bar = 100 μm), brown color reaction indicates cells that underwent apoptosis. Panel **D** shows cells at higher magnification (scale bar = 50 μm) and TUNEL—positive cells are indicated by arrows. Quantitative results are presented as a percentage of TUNEL-positive (panel **E**). The data are expressed as the mean ± SD; * *p* < 0.001 vs. the control.

**Figure 3:**
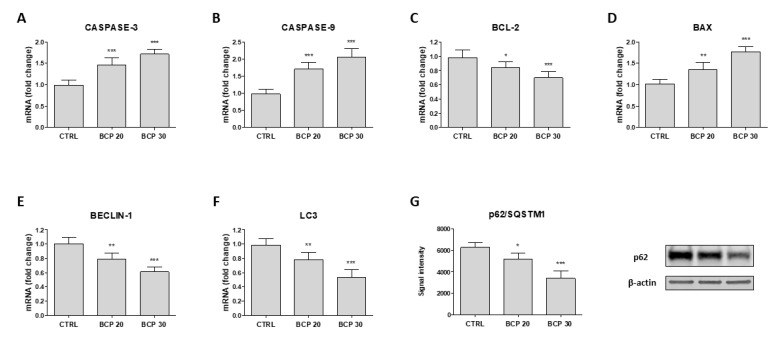
Effect of BCP on caspase-3 (**A**), caspase-9 (**B**), BCL-2 (**C**), BAX (**D**), BECLIN-1 (**E**), and LC3 (**F**) mRNA expression in U373 cells. (**G**) The graph represents p62/SQSTM1 protein expression. mRNA levels are calculated according to the 2^−ΔΔCt^ method as fold change relative to normal controls. Treatment with BCP significantly reduced p62 protein expression compared to the control; the blots were quantified by densitometric analysis. The data are expressed as signal intensity and as means ± SD; * *p* < 0.05, ** *p* < 0.01, *** *p* < 0.001 vs. the control.

The apoptotic cell death was first determined with Tunel assay. The results show that BCP treatment caused a marked increase in cell death through apoptosis following 24 h of treatment (Figure 2, panels A–D). In particular, the observed increase was of about 20% and 40% with 20 and 30 μg/mL BCP, respectively (Figure 2, panel E). Moreover, BCP treatment significantly increased caspase-3 and caspase-9 mRNA expression (Figure 3, panels A and B), thus providing evidence of apoptotic process activation. To further confirm these results, B-cell lymphoma (BCL)-2 and BCL Associated X (BAX) mRNA expression was examined, as markers of apoptosis. Consistent with the caspases results, BCP treatment induced a marked decrease in BCL-2 mRNA expression and increased BAX mRNA levels in U373 cells in a dose-dependent manner (Figure 3, panels C and D). In addition, Beclin-1, Microtubule-associated protein 1A/1B-light chain 3 (LC3) and p62/Sequestosome 1 (SQSTM1) were evaluated as markers of autophagy. Treatment with BCP resulted in a reduction of Beclin-1 and LC3 and p62/SQSTM1 expression compared to untreated U373 cells, indicating a possible switch of autophagy to apoptosis triggered by BCP (Figure 3, panels E–G).

### 2.3. BCP Plays an Anti-Inflammatory Activity through the Modulation of NF-κB and PPAR-γ 

**Figure 4:**
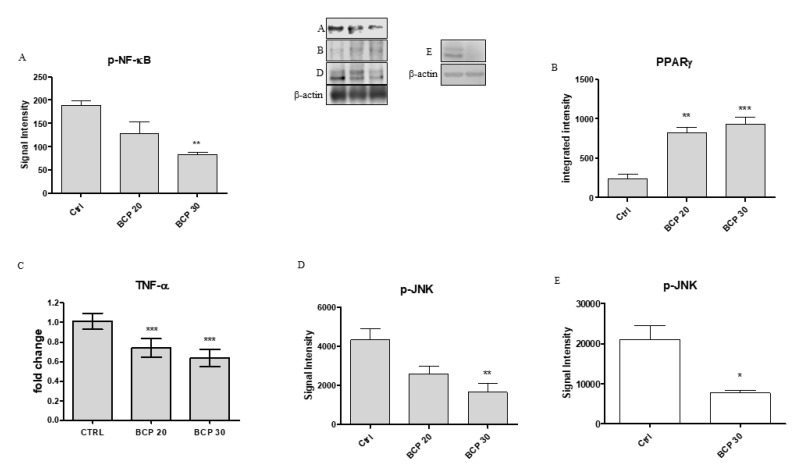
The graphs represent the protein expression of pNF-κB (**A**), PPARγ (**B**), pJNK (**D**,**E**) and mRNA expression of TNF-α (**C**) from U373 cells untreated and treated with BCP at the doses of 20 and 30 μg/mL and U87 cells (**E**) untreated and treated with BCP at the dose of 30 μg/mL. After the densitometric analysis, the data are expressed as signal intensity and as means ± SD. * *p* < 0.05, ** *p* < 0.01, *** *p* < 0.001, vs. the control.

In the present experiment, BCP treatment caused a significant reduction in phosphorylated NF-κB (p-NF-κB) and, in contrast, a significant increase in PPAR-γ at both doses of 20 and 30 μg/mL (Figure 4, panels A and B). In addition, Tumor Necrosis Factor alpha (TNF-α) mRNA expression was significantly reduced following BCP treatment at the dose of both 20 and 30 μg/mL, as a probable consequence of NF-κB reduction and PPAR-γ activation (Figure 4, Panel C).

### 2.4. BCP Carries Out an Anti-Proliferative Effect through Jun N-Terminal Kinase (JNK) Reduction 

We investigated the involvement of JNK in the anti-proliferative effects of BCP by Western Blot analysis. U373 cells showed high levels of phosphorylated JNK (p-JNK), whereas the treatment with BCP significantly reduced JNK expression, particularly at the concentration of 30 μg/mL (Figure 4, Panel D). Similar results were obtained using the p53 WT cells, U87, which revealed that BCP treatment at the dose of 30 μg/mL significantly reduced p-JNK expression compared to untreated cells (Figure 4, Panel E).

### 2.5. AM630, a CB2 Antagonist, Abrogates BCP Effects 

**Figure 5:**
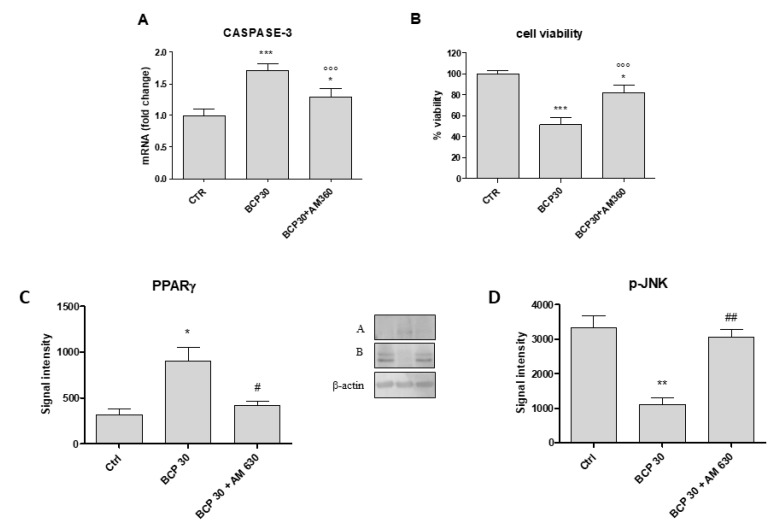
Graphs represent caspase-3 gene expression (**A**), cell viability (**B**), and the protein expression of PPARγ (**C**) and pJNK (**D**) in U373 cells untreated and treated with BCP at the dose 30 μg/mL and BCP (30 μg/mL) + AM630, cannabinoid receptor 2 (CB2) receptor antagonist. After densitometric analysis data are expressed as signal intensity and means ± SD. * *p* < 0.05 and ** *p* < 0.01 vs. the control and BCP+AM630; °°° *p* < 0.001 vs. BCP 30; ^#^ *p* < 0.05 and ^##^ *p* < 0.01 vs. BCP30. *** *p* < 0.001.

To evaluate if the anti-proliferative effect of BCP was mediated by CB2 activation in the U373 glioblastoma cell line, we treated cells with a specific CB2 receptor antagonist, AM630. The concomitant incubation of BCP at the dose of 30 μg/mL and AM630 reduced caspase-3 activation and partially restored cell viability (Figure 5, panels A and B). In addition, AM360 abrogated BCP effects on both PPARγ and JNK expression, restoring the protein levels of untreated cancer cells. Therefore, this experimental paradigm confirms the BCP mechanism of action, thus demonstrating that BCP effects were mediated by CB2 receptor (Figure 5, panels C and D).

### 2.6. β-Caryophyllene Reduces the Proliferation of Glioma Stem Cells and Inhibits Cell Cycle

Glioma stem cells (GSCs) were used to confirm BCP efficacy. The expression of two glioma stem cells-specific markers, CD133 and OCT4, were assayed in GSCs at the passage 6, the last culture passage used in the experiments, to confirm GSCs gene expression profile. Two specific bands of about 370 bp and 280 bp were detected for CD133 and OCT4 PCR products, respectively in agarose gel. Detection of specific bands following Western Blot analysis indicated that the cells were able to express specific stem cell markers (Figure S1). These results demonstrated that GSCs still maintained stem cell phenotypic profile at passage 6, therefore they were useful to evaluate BCP effects.

**Figure 6:**
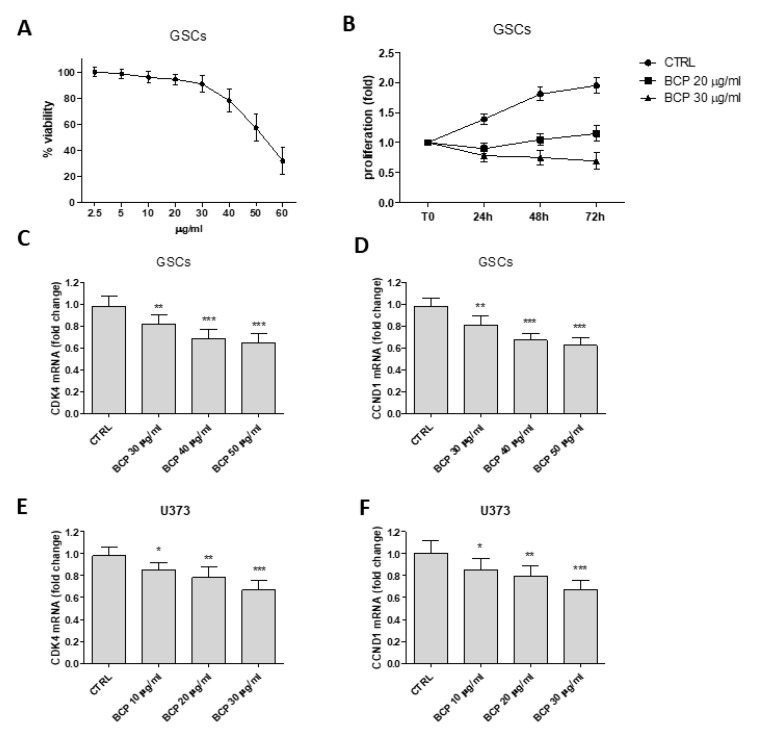
Dose response curve of cell viability assessed in GSCs treated with BCP (panel **A**). Values are expressed as percentage of reduction of viability with respect to the untreated control. Cell proliferation evaluation at T0, 24, 48 and 72 h in GSCs treated with BCP (panel **B**). Values are expressed as fold change of viability with respect to the untreated control at T0. Evaluation of CDK4 an CCND1 mRNA in GSCs and U373 cells treated with BCP at different doses (panels **C**–**F**). Data are calculated according to the 2^−ΔΔCt^ method as fold change relative to normal controls and expressed as means ± SD. * *p* < 0.05, ** *p* < 0.01, *** *p* < 0.001, vs. the control.

BCP significantly reduced cell viability and proliferation compared to untreated GSCs (Figure 6, panels A and B). BCP anti-proliferative effect was also confirmed by the inhibition of the cell cycle. In fact, BCP treatment significantly down-regulated Cyclin D1 and its cyclin-dependent kinase (CDK) 4 in both U373 glioblastoma cell line and in glioma stem cells (Figure 6, panels C–F). These results suggest that BCP regulates the uncontrolled cell cycle both in U373 glioblastoma cells and in the more resistant glioma stem cells.

## 3. Discussion

The identification of cannabinoid receptors in the central nervous system led to the hypothesis that targeting a cannabinoid receptor might be used as a therapeutic approach. High-grade gliomas, including glioblastomas, express high levels of CB2 receptors and their expression often correlates with malignancy [7]. The canonical signaling pathway for CBRs involves their coupling with Gi/0 and normally results in an overall inhibitory signal. Other pathways that can be activated by the binding of cannabinoids to CBRs involve the enzymes PI3 kinase, esphingomyelinase, and phospholipase C [13]. CB2R activation has been shown to mediate an inhibitory effect on the activation, cell motility and secretion of inflammatory mediators.

β-caryophyllene is a natural CB2 receptor agonist that potentially inhibits cell survival proteins [11] and also modulates the activation of NFκB and PPARγ; thus, it has been tested to reduce proliferation and activate apoptosis in glioblastoma. To investigate this issue, U373 and U87 cells were used: U373 is a human glioblastoma cell line with a p53 mutation in codon 273 of the p53 gene and cannot transactivate a reporter gene containing a p53-responsive transcriptional promoter, eventually causing tumor progression for p53 loss of function [14]. U87 cells are derived from glioma with a wild-type p53 and served as control to dissect out the effect of BCP in reducing cell proliferation in an abnormal (U373) and normal (U87) proliferative status. Following a short (24 h) and longer (72 h) incubation with BCP, a significant anti-proliferative effect was observed in both cell lines. In particular, BCP was only slightly more effective in U87, suggesting that the inhibitory effect is not mainly linked to p53 activation status. Scientific evidences described the role of glioma-derived stem-like cells in gliomas whose presence is a predictive factor of high malignancy [15,16]; they contribute to heterogeneity of tumors and are particularly resistant to therapies. Therefore, GSCs were used in this study to confirm BCP anti-proliferative efficacy. BCP reduced cell viability in glioma-derived stem-like cells, thus demonstrating that this therapeutic approach might also be effective in conditions of resistance. BCP also modulated cell cycle both in GSCs and in the U373 cell line, reducing Cyclin D1 and CDK4 expression. This is a relevant piece of data since cell cycle is uncontrolled in tumors and targeting cell cycle, using BCP, may be an interesting therapeutic approach in aggressive cancers such as glioblastomas. The results obtained in GSCs are therefore of paramount importance because they confer to BCP a more powerful translational potential. However, it could be argued that GSCs might lose their characteristic phenotype, thus weakening our scientific message. To check for this bias, we evaluated two glioma stem-cell-specific markers, CD133 and OCT4, that were studied in GSCs at the passage 6, the last culture passage used in our experiments. The results clearly show that GSCs still maintained their stem cell phenotypic profile at passage 6, thus ruling out the loss of aggressiveness stigmata that make it the closest experimental paradigm to the clinical setting.

The inhibitory effect of BCP led us to hypothesize the involvement of a pathway that is, at least in part, unrelated to cell survival, as indicated by the reduced expression of p-JNK both in U373 and U87 cell lines, an effect that was reverted by using the CB2 receptor antagonist AM630. Thus, the apoptotic activation seems to be related to the activation of PPARγ and this might be true considering PPARγ as a pro-differentiating factor whose increase led the cells to a more differentiated status, cells that fail differentiation consequently die for the activation of the apoptotic process. Our results are in agreement with this hypothesis; in fact, the increased expression of effector caspases 3 and 9, and BAX has been observed as early as 24 h following BCP treatment also confirmed by Tunel assay. In addition, the treatment with AM630 was able to restore cell viability and reduced caspase-3 expression, confirming the involmement of CB2 receptor activation in the trigger of apoptosis. 

Anti-apoptotic protein Bcl-2 may bind Beclin-1, whose activation regulates the autophagic process. Autophagy promotes cell survival in cancers, thus increasing tumor growth and invasiveness [17]. During autophagy, autophagosomes are produced to embed intracellular constituents and organelles, such as mitochondria. In this process, LC3 is engaged in the assembly and disassembly of microtubules [18,19]; in particular, LC3-I is converted to LC3-II through phosphatidylethanolamine (PE) conjugation and p62/SQSTM1 is considered as the LC3 substrate, thus facilitating selective degradation in the autophagy process.

In the present study, Beclin-1, LC3 and p62/SQSTM1 decrease was observed in U373 BCP-treated cells. These results demonstrate that the autophagy process was reduced following the treatment with BCP compared to untreated U-373 cancer cells and allow us to hypothesize a switch of autophagy to apoptosis, further confirming that apoptotic process was activated following the treatment with BCP. Therefore, this CB2 agonist may be considered as an anti-proliferative and protective molecule against malignant and aggressive cancers such as glioblastoma.

Apart from a direct killing effect on tumor cells, cannabinoids can also induce a significant reduction of inflammation, thereby inhibiting upstream and downstream molecules involved in inflammatory process. The curative anti-inflammatory effect of BCP was demonstrated by studying the transcription factor NF-κB, the activation of which causes the increase in pro-inflammatory cytokines, such as TNF-α. In this experimental approach, U373 cells highly expressed NF-κB, whereas BCP treatment significantly reduced its expression. In addition, previous studies have indicated a possible crosstalk between the CB2 receptor and PPAR-γ [12,20]. The PPAR-γ receptor activation reduces pro-inflammatory cytokine release, thus exerting an anti-inflammatory effect [21]. The obtained data demonstrate that BCP may also act through PPAR-γ; in fact, the treatment of glioblastoma cells with BCP significantly increased the PPAR-γ receptor. As expected, the pro-inflammatory cytokine TNF-α was highly expressed in glioblastoma cells, and its expression was significantly reduced following BCP treatment, probably as a consequence of NF-κB reduction and PPAR-γ activation.

Fascinatingly, it has been observed that the use of CB2 agonists may cause an accumulation of TNFα transcripts and a decrease in protein expression [22,23]. Moreover, the JNK pathway blockade may inhibit TNFα [24], but TNFα, at the same time, activates MAPK pathways, such as ERK1/2, JNK, and p38, which contributes to inflammation and cell proliferation and migration. In particular, the JNK pathway is involved in different processes, such as cell proliferation, differentiation, inflammatory response, cell survival and death. 

Interestingly, scientific evidences described the JNK role in autophagy through Bcl-2 and Bcl-xL phosphorylation to active Beclin-1 [25]. In particular, as previously described, Bcl-2 and Beclin-1 reduction may be a direct consequence of JNK decrease [26]. Therefore, the results discussed so far support the idea that BCP may also regulate apoptosis and autophagy mechanisms not only through a direct modulation of CB2 receptors but also by JNK modulation. Several cancer therapeutics do not cross the blood–brain barrier (BBB), and other biochemical and physical factors such as the high brain efflux index (BEI), the electrostatically charged and anisotropic brain extracellular space (ECS) may block drugs crossing through the BBB. However, previous studies have already demonstrated that BCP is a small and lipid-soluble molecule (204.36 Da) that has the ability to cross the BBB [27,28]. This preliminary in vitro study put the basis for the possible use of BCP for the treatment of glioblastomas but an in vivo model and later clinical studies will be needed to confirm the data obtained so far. 

## 4. Materials and Methods

### 4.1. Cell Cultures

U-373 MG (Uppsala; p53 mutant) and U-87 MG (p53 wild type), human glioblastoma astrocytoma cell lines derived from a malignant tumor by explant technique, were provided by ECACC cell collection (Salisbury, UK). The cells were cultured in EMEM (Eagle’s Minimal Essential Medium) supplemented with 2 mM glutamine, 1% non-essential amino acids (NEAA), 1 mM sodium pyruvate, 10% FBS (fetal bovine serum), 1% antibiotic mixture (Sigma-Aldrich, St. Louis, MO, USA), in a 5% CO_2_ humidified incubator at 37 °C. Human glioma stem-like cells (GSCs) were purchased by AcceGen Biotechnologies (USA) and cultured in the specific human glioma cancer cell medium provided by the same company. The media were renewed every 2 days and confluent cells were trypsinized, subdivided, and re-plated. In these experiments, U373 and U87 cell lines were used from passage 5 to 10, and the GSCs were used from passage 3 to 6. In addition, the expression of two genes associated with GSCs, CD133 and OCT4 (octamer-binding transcription factor 4), was examined at the passage 6 by RT-PCR; Western Blot analysis was also performed to evaluate protein expression. The amplified PCR products were separated on 1.5% agarose gels and visualized by ethidium bromide staining.

### 4.2. Cell Treatment

U373 cells were cultured in six well culture plates at a density of 2.5 × 10^5^ cells/well. Sixteen hours after seeding, a set of plates were treated with β-caryophyllene (BCP) (Sigma-Aldrich, USA), dissolved in DMSO (20 mg/mL), at doses of 20 and 30 μg/mL (the calculated IC 50 was about 29 μg/mL). In a further set of plates, a specific CB2 antagonist, AM360 (Tocris Biosciences, Abingdon, UK), was added at the dose of 100 nM 2 h before the treatment with BCP (30 μg/mL). The cells underwent biochemical and molecular evaluation 24 h after the treatments. 

### 4.3. MTT Assay 

Cell viability was evaluated by MTT assay. U373, U87 and GSCs cells were grown and treated with BCP at doses of 2.5, 5, 10, 20, 30, 40, 50 and 60 μg/mL in a 48 well-plate at a density of 4 × 10^5^ cells/well for 24 h to evaluate the cytotoxic effect. In a further set of plates, cells were seeded 2 × 10^5^ cells/well and treated with BCP at doses of 5 and 10 μg/mL for 72 h to evaluate the antiproliferative effect. The tetrazolium dye MTT 3-(4,5-dimethylthiazol-2-yl)-2,5-diphenyltetrazolium bromide (Sigma Aldrich, Milan, Italy) was dissolved in sterile filtered PBS (5 mg/mL) and 40 µL of the mixture was added into each well 3 h before the end of the 24 h of incubation. Medium was removed and the insoluble formazan crystals were dissolved with dimethyl sulfoxide (DMSO; 1 mL/well). Absorbance was measured at 550 nm using a spectrophotometer (Biospectrometer basic, Eppendorf, Hauppauge, NY, USA). The results were expressed as % of cell viability compared to untreated cells and reported as means and SD.

### 4.4. Tunel Assay

The DeadEnd™ Colorimetric TUNEL System was used to detect apoptotic cells in situ in cultured cells, according manufacturer’s instructions. The systems end-label the fragmented DNA of apoptotic nuclei using a modified Tunel method. Briefly, U373 were seeded on Lab-Tek^®^ Chamber Slides (10^4^ cell/well) and treated with BCP at doses of 20 and 30 μg/mL for 24 h. Then, cells were fixed in 4% paraformaldehyde solution in PBS for 25 min at room temperature. After permeabilization with 0.2% of Triton–X100 solution in PBS, for 5 min, and rinsing with PBS, cells were incubated with a mix containing biotinylated nucleotide and Terminal Deoxynucleotidyl Transferase, Recombinant, (rTdT) enzyme for 60 min at 37 °C. After rinsing, slides were incubated with a streptavidin HRP for 30 min at room temperature. Following the addition of the chromogen diaminobenzidine (DAB), apoptotic nuclei are stained dark brown and visualized with a light microscope (20× objective, Leica ICC50).

A total of 20 representative images were investigated for each sample and the number of apoptotic cell was determined by counting the total number of nuclei per image area and the TUNEL-positive nuclei. The results were expressed as % of apoptotic cells. 

### 4.5. RNA Isolation, cDNA Synthesis, and Real-Time Quantitative PCR Amplification

Total RNA was isolated from U373 cells for RT-qPCR analysis using a Trizol Reagent Kit (Life Technologies, Monza, Italy). The first strand of cDNA was synthesized from 2.0 μg total RNA using a high capacity cDNA Archive kit (Applied Biosystems, Carlsbad, CA, USA). β-actin mRNA was used as an endogenous control to allow for the relative quantification. RTqPCR was performed to evaluate the gene expression of BCL-2, BAX, caspase-3, caspase-9, TNF-α, eclin-1, CDK4 and CCND1 using Power Up Sybr Master Mix (Applied Byosystems, CA, USA) and a QuantStudio 6 Flex Real-Time PCR System (Applied Biosystems, CA, USA). The amplified PCR products were quantified by measuring the calculated cycle thresholds (CT) of target genes and β-actin mRNA. After normalization, the mean value of the normal control target levels was chosen as the calibrator and the results were expressed according to the 2^−ΔΔCt^ method, as a fold change relative to normal controls. The oligonucleotide sequences of the used primers are reported in Table 1.

### 4.6. Western Blot Analysis

The cells were homogenized in RIPA buffer (25 mM Tris/HCl, pH 7.4; 1.0 mM EGTA; 1.0 mM EDTA) with 1% of NP40, 0.5% of phenyl methylsulfonyl fluoride (PMSF), aprotinin, leupeptin and peptastatin (10 μg/mL each) to perform protein extraction. The lysates were centrifuged at 1500× *g* for 15 min at 4 °C and the supernatant was collected for protein determination using a specific kit (Bio-Rad DC; Bio-Rad, Richmond, CA, USA). The samples were denatured in reducing buffer (62 mM Tris pH 6.8, 10% glycerol, 2% SDS, 5% β-mercaptoethanol, 0.003% bromophenol blue) and the proteins were separated by electrophoresis on an SDS polyacrylamide gel (10%). Following electrophoresis, the samples were transferred onto a PVDF membrane (Amersham, Little Chalfont, UK) in a transfer buffer (39 mM glycine, 48 mM Tris pH 8.3, 20% methanol) at 200 mA for 1 h. The membranes were incubated with 5% non-fat dry milk in TBS-0.1% Tween for 1 h at room temperature, washed 3 times in TBS-0.1% Tween, and incubated with primary antibodies pNF-κB, PPARγ, p-JNK, p62/SQSTM1 (Cell Signaling, Danvers, MA), CD133 and OCT4 (Abcam, Cambridge, UK) diluted in TBS-0.1% Tween overnight at 4 °C. The day after and following 3 washes with TBS-0.1% Tween, the membranes were incubated with secondary peroxidise-conjugated goat anti-mouse and anti-rabbit antibodies (KPL, Gaithersburg, MD, USA) for 1 h at room temperature. After washing, the membranes were analyzed by the enhanced chemiluminescence system (LumiGlo reserve; Seracare, Milford, MA, USA). The protein signal was detected and quantified by scanning densitometry using a bio-image analysis system (C-DiGit, Li-cor, Lincoln, NE, USA). The results were expressed as relative integrated intensity. β-actin (Cell Signalling, Danvers, MA, USA) was used to confirm equal protein loading. Whole blot can be found at Figure S2.

### 4.7. Statistical Analysis

The results are expressed as means ± standard deviation (SD). The values reported are the result of at least five experiments performed in duplicate. All assays were repeated three times to ensure reproducibility. Different groups were analyzed by *t*-test and one-way ANOVA with Tukey’s post-test for intergroup comparisons. A p value less than 0.05 was considered significant. Graphs were set using GraphPad Prism (Version 5.0 for Windows).

## 5. Conclusions 

BCP may be considered as an inhibitor of cell proliferation mechanisms in invasive cancers such as glioblastoma thanks to the modulation of the cannabinoid system. Despite the fact that the translational potential of this study might be related to the use of human cancer cells, further investigations will be carried out to confirm the obtained in vitro results.

## Figures and Tables

**Table 1:** Primer sequences used for RT-qPCR.

Gene	Forward Primer 5′-3′	Reverse Primer 5′-3′
ACTB	TTGTTACAGGAAGTCCCTTGCC	ATGCTATCACCTCCCCTGTGT
CASPASE-3	CTGAGGCATGGTGAAGAAGGA	GTCCAGTTCTGTACCACGGCA
CASPASE-9	TGCGAACTAACAGGCAAGCA	GTCTGAGAACCTCTGGTTTGC
BCL-2	GAGGATTGTGGCCTTCTTTGAG	AGCCTCCGTTATCCTGGATC
BAX	GGACGAACTGGACAGTAACATG	GCAAAGTAGAAAAGGGCGACA
TNF-α	GATAGATGGGCTCATACCAGGG	TCTTCAAGGGCCAAGGCT
Beclin-1	TGAGAGACTGGATCAGGAGG	CGCATCTGGTTTTCAACACTC
LC3	AAGGCGCTTACAGCTCAATG	CTGGGAGGCATAGACCATGT
CDK4	AGCCGAAACGATCAAGGAT	GCTTGACTGTTCCACCACTTG
CCND1	CACCTTATTCATGGCTGAAGTC	ACAAACCTCCACTGGATGGT
CD133	CTTGGCTCAGACTGGTAAATCC	CCACTTTCTCACTGATAGAG
OCT4	AGAAGGATGTGGTCCGAGTG	GCACCTCAGTTTGAATGCATG

## Supplementary Materials

The following are available online at https://www.mdpi.com/2072-6694/12/4/1038/s1, Figure S1: Evaluation of specific stem cell gene expression patterns in GSCs at passage 6, Figure S2: Whole blot (uncropped blots) for figures in main text.
