# Supplementary material for: β-Caryophyllene Inhibits Cell Proliferation through a Direct Modulation of CB2 Receptors in Glioblastoma Cells
Source: Cancers (Basel). 2020 Apr 23;12(4):1038. doi: 10.3390/cancers12041038 (PMC7226353; doi:10.3390/cancers12041038)
Supplement: Supplementary file 1 [file cancers-12-01038-s001.pdf]

Supplementary Materials: β-Caryophyllene Inhibits Cell Proliferation through a Direct Modulation of CB2 Receptors in Glioblastoma Cells

Natasha Irrera, Angela D'Ascola, Giovanni Pallio, Alessandra Bitto, Federica Mannino, Vincenzo, Arcoraci, Michelangelo Rottura, Antonio Ieni, Letteria Minutoli, Daniela Metro, Mario Vaccaro, Domenica Altavilla and Francesco Squadrito

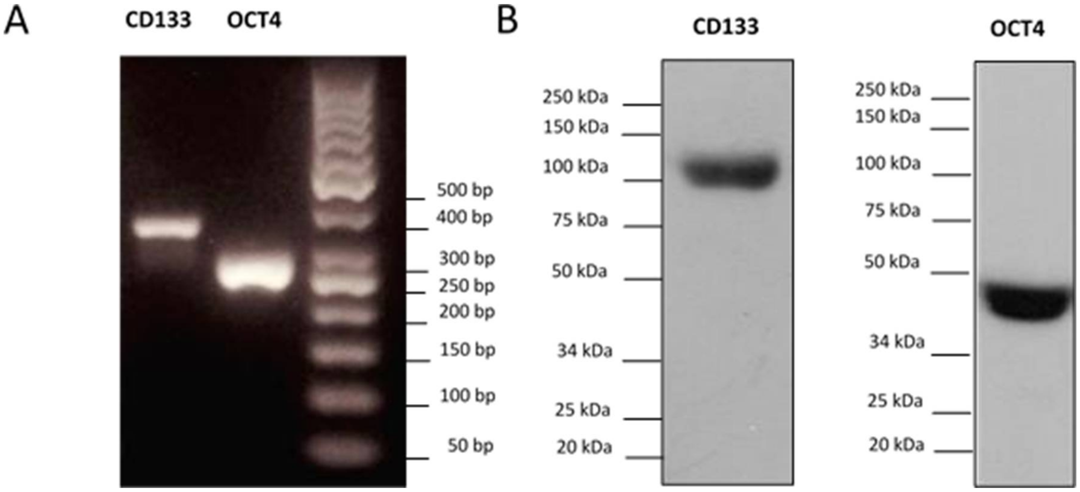

Figure S1. Evaluation of specific stem cell gene expression patterns in GSCs at passage 6. **A**) Agarose gel electrophoresis of CD133 and OCT4 RT-PCR products. The specific DNA bands were detected under UV light after staining with ethidium bromide. **B**) Protein expression of CD133 and OCT4. Data showed expression of these stem cell markers in GSCs.

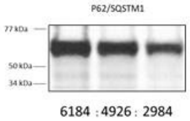

Figure 3

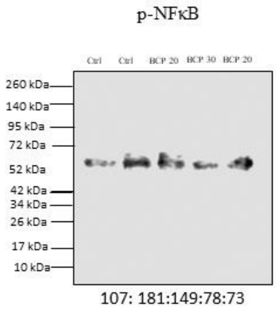

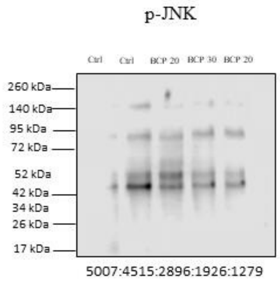

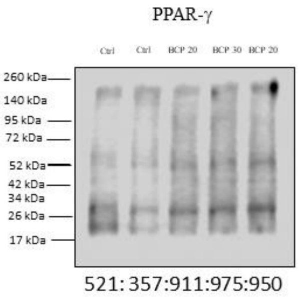

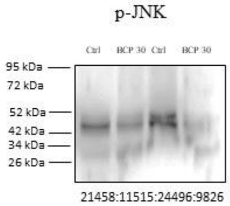

Figure 4

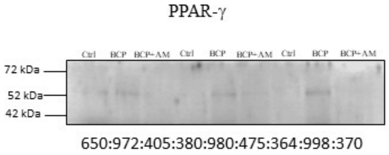

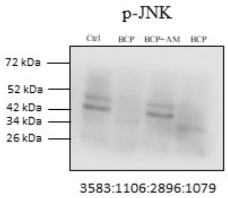

Figure 5

Figure S2. Whole blot (uncropped blots) for figures in main text.

© 2020 by the authors. Submitted for possible open access publication under the terms and conditions of the Creative Commons Attribution (CC BY) license (http://creativecommons.org/licenses/by/4.0/).
